# Advances and safe use of energy devices in lung cancer surgery

**DOI:** 10.1007/s11748-022-01775-w

**Published:** 2022-02-02

**Authors:** Takahiro Homma

**Affiliations:** Department of General Thoracic and Cardiovascular Surgery, Graduate School of Medicine and Pharmaceutical Sciences, University of Toyama, 2630 Sugitani, Toyama, Toyama 930-0194 Japan

**Keywords:** Energy device, Monopolar device, Bipolar device, Ultrasonic energy device, Lung cancer

## Abstract

**Objectives:**

A clear understanding of energy devices would help achieve high effectiveness and safety and guide the selection of devices. The present review aimed to elucidate the efficacy and adverse events of energy devices in lung cancer to guide the selection of appropriate devices depending on the situation.

**Methods:**

Four major databases were searched electronically for relevant articles published until 16 April 2021. The reference lists of the identified papers were examined. We excluded (1) irrelevant studies, (2) manuscripts published in languages other than English and Japanese, (3) duplicates, and (4) studies for which the full text was not available in the databases. The results and key information obtained were summarized by means of a narrative approach.

**Results:**

A total of 78 papers were included in the review and these were categorized according to the main topic of investigation as follows: (1) electrosurgery-related injuries, (2) fundamentals of electrosurgery, (3) monopolar devices, (4) bipolar electrosurgical devices, (5) ultrasonic energy devices, (6) energy devices in lung cancer surgery, (7) operating room fire risks, and (8) basic principles of surgery.

**Conclusions:**

Understanding energy devices could help us use them in a more effective and safer manner. Knowledge of their selection criteria (suitability), merits, risks, and safety precautions relevant to each process of lung cancer surgery could guide appropriate selection.

**Supplementary Information:**

The online version contains supplementary material available at 10.1007/s11748-022-01775-w.

## Introduction

The standard treatment for clinical stage I–II non-small cell lung cancer is surgery. Based on the results of a clinical trial conducted in 1995, lobectomy and lymph node dissection of the hilar and mediastinum are the standard surgical procedures for non-small cell lung cancer [1]. Various minimally invasive approaches have been developed over the last decade, ranging from conventional thoracotomy to video-assisted thoracoscopic surgery (VATS), robot-assisted thoracoscopic surgery, uniportal VATS, and subxiphoid approach [2–4].

In surgical treatment for lung cancer, energy devices and automatic suture devices have significantly contributed to the evolution and safety of surgical treatment regardless of procedures or approaches. As for energy devices, monopolar devices, advanced bipolar devices, and ultrasonic energy devices are frequently used. The improved vascular sealing function of advanced bipolar devices and ultrasonic energy devices is remarkable. Ligation, a classical hemostasis method, is no longer necessary for a small vessel. While they improve the quality and safety of surgery, their misuse can put the patients at risk. Therefore, it is desirable to use them only after understanding their principles and basic mechanisms.

## Methods

This review covers different energy devices, electrosurgery-related injuries, energy device education, and operating room fire risks with the aim of providing an overview of advances in and the safe use of energy devices. In particular, we focused on the effects, adverse events, and better choice of energy devices in lung cancer surgery.

To identify the effects and adverse events of energy devices, a literature search within the PubMed, Medline, Scopus and Web of Sciences databases were searched electronically from their inception up to 16 April 2021 (Supplement file). Papers published until the date of the review that contained these terms in the abstract were selected.

Reference lists of the identified papers and relevant manuscripts were examined. The titles and abstracts’ information were selected for subject importance. Studies that were not definitively excluded on the basis of abstract information were also selected for full-text screening. The full text of all relevant researches to evaluate the possibility of inclusion was examined. The criteria of exclusion were as follows: (1) studies not focused on the topic selected, (2) papers in a language other than English and Japanese, (3) duplicates, and (4) studies not available from libraries for full-text assessment. The results and key information obtained were summarized by means of a narrative approach.

The publications indexed as articles, proceedings papers or reviews were reviewed, including the references of the publications to identify additional relevant articles; finally, a total of 77 papers were included in the review. The papers were decided to categorize these articles based on theoretical considerations.

## Electrosurgery-related injuries

Surgical medicine has evolved using hemostatic methods. Electrosurgical devices have been in use for approximately 100 years [5] (Ref; 1. Evolutions and revolutions in surgical energy). Over recent years, while advanced equipment has been developed and clinically introduced almost every year, such equipment has been utilized without systematic education, and a lack of knowledge about energy devices has been revealed, irrespective of experience [6].

Energy devices cause various adverse events, ranging from burns and organ damage to operating room fires; nonetheless, these events are not well known. Electrosurgery-related injuries are estimated to occur at an incidence of approximately 40,000/year [7] or approximately 1–2 per 1000 operations during laparoscopy [8]. The impact is significant, with paid claims of nearly 600 million USD for electrosurgical injuries in 1999 [7]. Moreover, 18% of surgeons have reportedly experienced electrosurgery-related injuries, and 54% have known a colleague who experienced such an injury [9].

The requirement for systematic education has increased against the backdrop of electrosurgery-related injuries in North America. Led by the Society of American Gastrointestinal and Endoscopic Surgeons (SAGES), surgeons, operating room nurses, anesthesiologists, electrical engineering specialists, educational specialists, and statistics experts have organized a team to develop FUSE, a specialized education program for the safe use of energy devices [5] (Ref; Introduction). The contents of FUSE are wide-ranging, focusing on the principles and safe use of monopolar devices, bipolar devices, ultrasonic energy devices, radiofrequency devices, microwave ablation, cryoablation, and endoscopic energy devices. Textbooks and e-learning are the modes of learning, and FUSE is also used as a qualification test and can be certified.

In Japan, the Pharmaceuticals and Medical Devices Agency (PMDA) has a mechanism to call attention to adverse events that can occur with medical devices and drugs that are disseminated worldwide [10]. Nevertheless, electrosurgery-related injuries still occur. Systematic education is necessary for their elimination. Therefore, lectures regarding the safe use of energy devices have been given to medical staff and students by incorporating material regarding these devices into the syllabus [11, 12].

## Fundamentals of electrosurgery

Electrosurgery uses radiofrequency (RF) alternating current (AC) to increase the intracellular temperature. In an AC, the polarity of intracellular ions and/or electrons rapidly and regularly fluctuates, and this mechanical energy changes to thermal energy, increasing the intracellular temperature near the tip of the RF energy device. If the cellular or tissue temperature is maintained at 50 °C for 6 min, cell death occurs. If the temperature reaches 60–100 °C, cell death instantaneously occurs along with cellular desiccation and protein coagulation. When the temperature reaches 100 °C, cells are vaporized through liquid–gas conversion to steam [5] (Ref; 2. Fundamentals of Electrosurgery Part I).

Intracellular thermal changes using AC are affected according to Ohm’s law [13]. Electrosurgery generators (or electrosurgical units; ESUs) convert low-frequency AC into higher frequency outputs and adjust current (*I*), voltage (*V*), and current time (duty cycle) to achieve various modes such as “*coagulation*” and “*cut*.” The impact of RF energy on tissues is related to the waveform of the ESU output [12, 14].

The mode depends on the current time and peak voltage. According to power (*W*) = voltage (*V*) × current (*I*), even with the same output, if the current time is long (continuous wave), the voltage tends to be low, and if the current time is short (interrupted wave), the voltage tends to be high. Low-voltage continuous outputs correspond to “*cut*” and “*soft coag*” modes. This results in a predictable zone of coagulation with higher quality and consistency. Therefore, low-voltage continuous outputs are generally the most effective for sealing blood vessels. The outputs of advanced bipolar devices are predetermined by low-voltage continuous outputs. However, the “*soft coag*” mode does not have a cutting effect, even under a focusing current density. In contrast, high-voltage interrupted outputs correspond to “*fulgurate*,” “*coag*,” and “*spray*” modes. In general, “*spray*” has the lowest duty cycle and highest peak voltage. These modes create a superficial and inhomogeneous zone of desiccation and coagulation that is unsuitable for vessel sealing.

## Monopolar devices

Monopolar systems include two separate instruments in the circuit: an active electrode and a dispersive electrode. Monopolar systems include the entire patient in the circuit. In other words, it is not possible to know exactly where the current flow in the body. This recognition is essential for understanding the mechanisms of electrosurgery-related injuries. The thermal effect is proportional to the current density squared [5] (Ref; 2. Fundamentals of Electrosurgery Part I). Adverse events can occur when the current density increases outside the tip [15].

### Active electrodes

Even if the tip of the active electrode does not make contact with the intended tissue, the effect may occur at an unexpected site. Inadvertent activation is a typical example. It is important to contact with the target before activation, avoid using a foot pedal that may cause inadvertent activation, and return the device to a nurse or a protective holder after use. The tip of the monopolar device should never be placed on the skin of the patient or the drapes over the skin when not in use.

### Insulation failure of reusable instruments

**Fig. 1:**
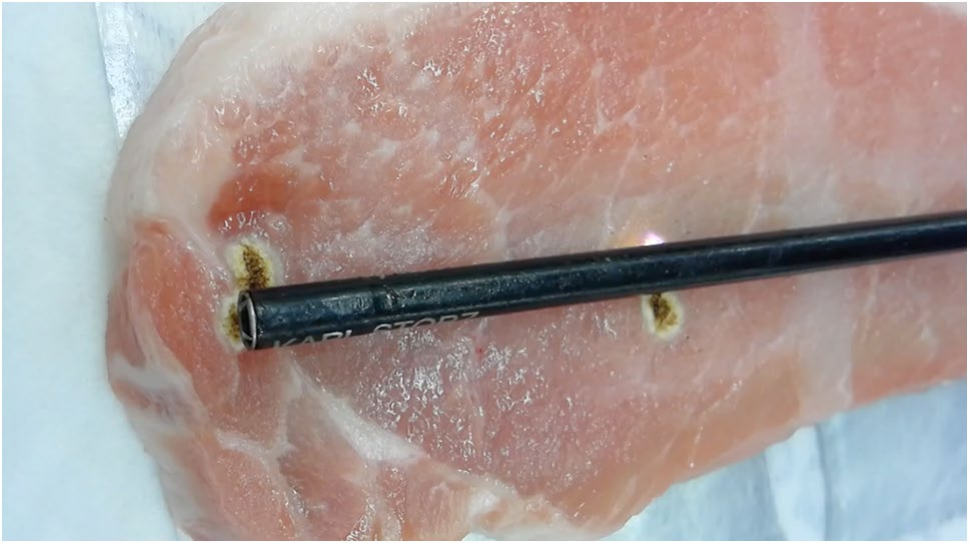
Forceps with insulation failure and energizing at a location other than the tip (arrow)

All insulated electrosurgical instruments should be regularly monitored for insulation breaks (Fig. 1). Robotic forceps are one of the types of reusable forceps used in surgery; however, it has been reported that 81.6% of these forceps had insulation failure after the tenth use [16, 17]. It is necessary to recognize that insulation failure can occur at any time.

### Staple line bleeding

**Fig. 2:**
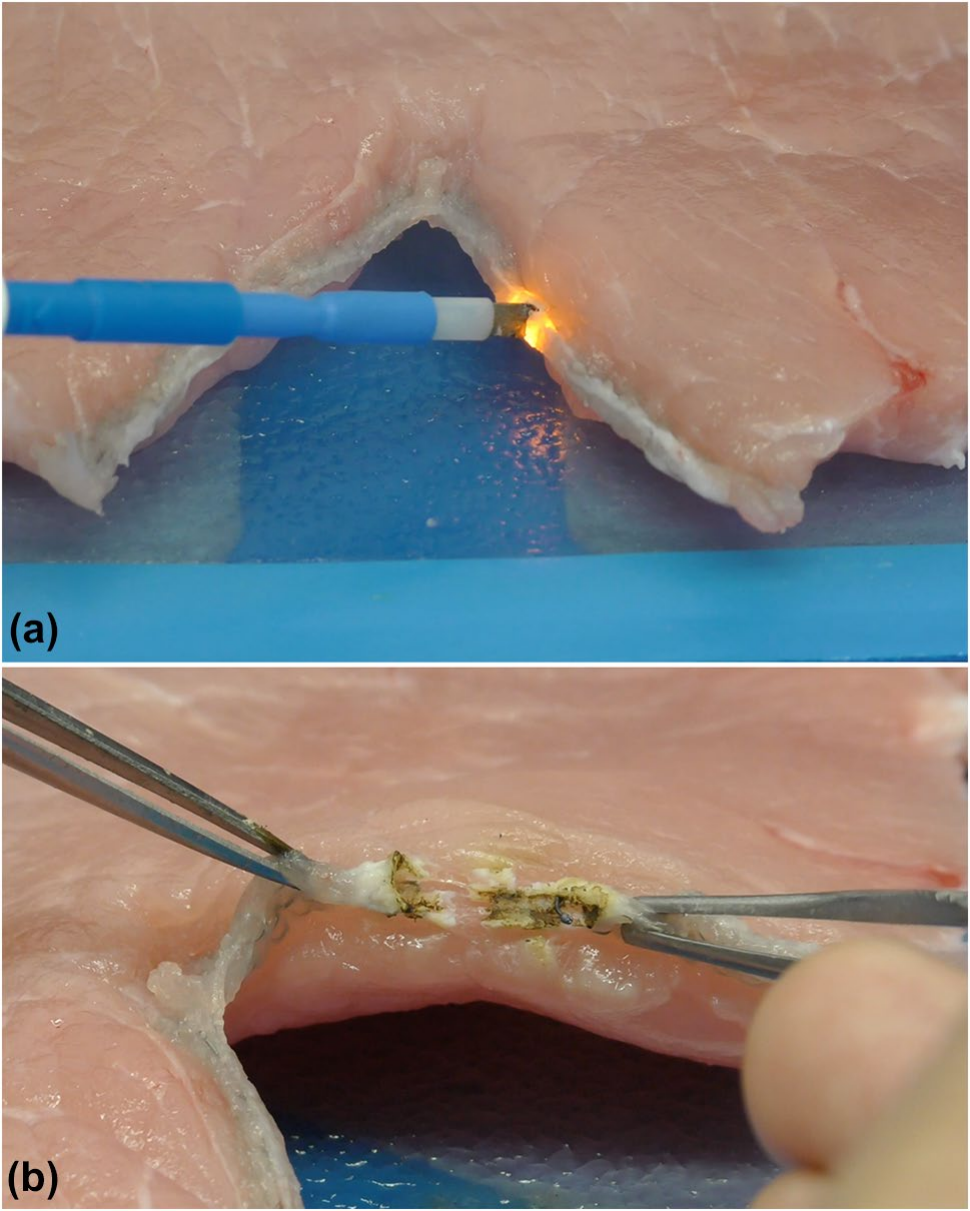
Energization experiment on staple line. **a** The current has the property of flowing to a place with low resistance. **b** Melted staple line

Monopolar electrosurgery is risky for the hemostasis of staple line bleeding. The current has the property of flowing to a place with low resistance. The row of metal staples creates a low-resistance pathway that allows the current to increase dramatically (metal-to-metal arching), and sufficient heat can be generated in the pathway to melt the exposed staple, which occurs at 1000 °C. Given a continuous arc between the active electrode and staple line or metal clips, delayed and unrecognized tissue breakdown and anastomotic leak may occur in the tissue around the metal [5] (Ref; 4. The Art and Science of Monopolar Electrosurgery). Thus, for bleeding from the bronchus or lung resection staple lines, monopolar devices should not be used [18], and sutures are preferred (Fig. 2).

Adverse events do not always occur under these conditions; thus, it is important to recognize risky behaviors. Preventative measures include activation after contact with the intended tissue, low output, low voltage, short time activation, activation in a closed circuit, regular monitoring for insulation failure, and the direction and location of the dispersive electrode.

## Bipolar electrosurgical devices

**Fig. 3:**
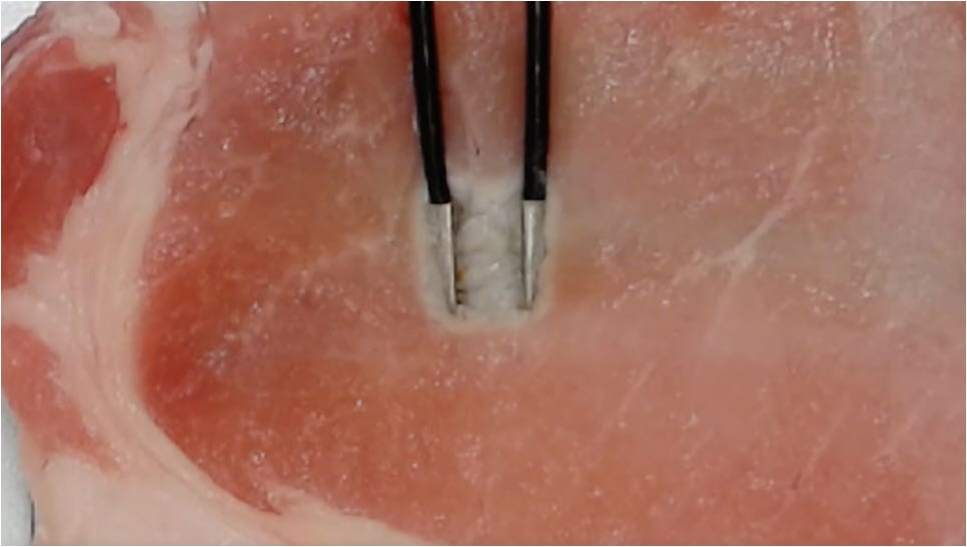
Collateral damage caused by a conventional bipolar device. A thermal effect was observed not only between the two electrodes but also around them

Bipolar devices are designed with both electrodes positioned on the same surgical device [5] (Ref; 5. Bipolar Electrosurgical Devices). When compared with monopolar devices, bipolar devices can control the flow of current through only the targeted tissue area and reduce the risk of remote electrosurgery-related injuries. However, the clinical effects of conventional bipolar devices are left to visual judgment. The degree of compressed tissue also depends on the tactile sensation of the surgeon. Therefore, conventional bipolar devices have risks in terms of improper or over-compression or activation of the tissue which may result in collateral damage (Fig. 3) and ineffective sealing.

New bipolar devices have been developed to overcome these limitations. So-called “advanced bipolar devices” such as LigaSure™ or EnSeal™ incorporate sophisticated microprocessors and feedback systems that monitor impedance and temperature and automatically adjust the delivery of the current to ensure adequate tissue sealing while minimizing collateral damage. It is also called a “vessel sealing device” or “vessel sealer” because the accuracy of sealing has improved dramatically. Advanced bipolar devices provide audible signals that indicate when adequate coagulation has been achieved. When energized with staples, the impedance deviates from the normal tissues; thus, the mechanism is safer because it does not erroneously energize. Advanced bipolar devices are capable of constantly achieving hemostatic tissue seals in vessels up to 7 mm in diameter.

As the temperature is controlled below 90 °C, the effect around the sealing tissue is minimal; nevertheless, collateral damage is not eliminated. Although the temperature of LigaSure™ is lower than that of ultrasonic devices, the thermal spread of LigaSure™ is significantly greater than that induced by ultrasonic devices [19]. Moreover, using LigaSure™ around nerves may have a risk of paralysis if the distance to the nerve is less than 3 mm.

**Fig. 4:**
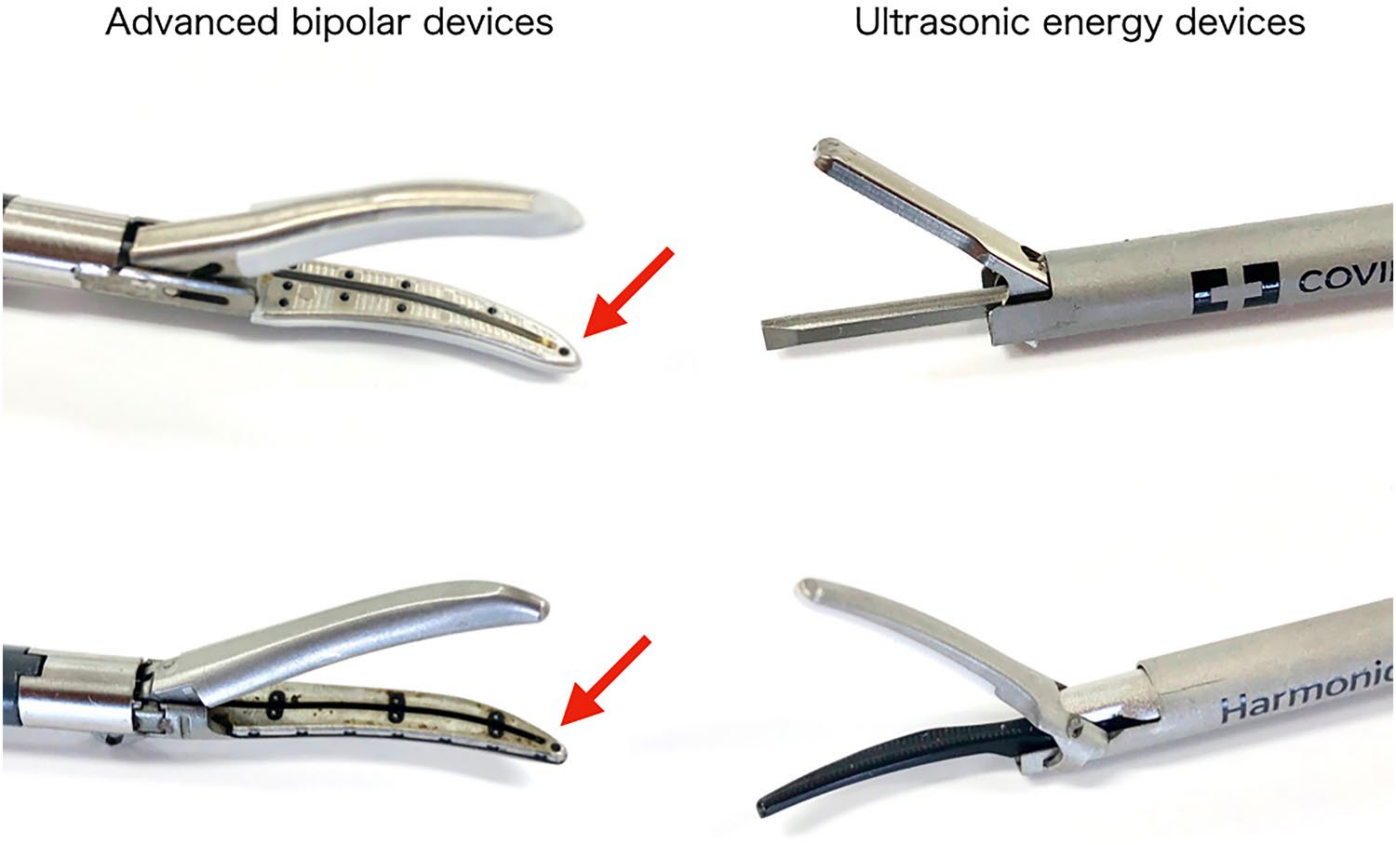
An advanced bipolar device (left) has a cutting line (groove indicated by the red arrow) from the bottom to a short distance before the tip. On the other hand, an ultrasonic energy device (right) can be cut from the bottom to the top (all the sandwiched parts)

Advanced bipolar devices can be used regardless of the tip direction and cannot cut at the tip. Surgeons need not pay much attention to contact with vital structures after activation, although ultrasonic devices may drill into the tissue (Fig. 4). Due to the better sealing function, care must be taken not to seal unintended layers (Table 1).

**Table 1:** Comparison of advanced and conventional bipolar devices

	Conventional	Advanced
Activation	Self-regulation	Auto-regulation
Effective area	Point	Plane
Sealing effect	Weak	Strong
Cutting effect	No	With a built-in knife
Lateral thermal spread	Yes, but depends on the activation time	
Handling	No activation unless sandwiching	Activation without sandwiching
	Coagulation only	Dissection and cut are also possible
Cost	Cheaper	Higher
Reusable	Yes	No

## Ultrasonic energy devices

Ultrasound refers to sound waves that are greater than 20 kHz. Ultrasound for diagnostic imaging wave frequencies ranges from 2 to 18 MHz. Ultrasonic energy devices such as the Harmonic™ or Sonicision™ contain piezoelectric ceramic discs that convert electrical energy into mechanical motion that is transferred to the shaft where it is amplified by silicon nodes [5] (Ref; 7. Ultrasonic Energy Systems). The active blade of the ultrasonic energy device vibrates at a rate of 23–55 kHz. This effect is caused by the mechanical interaction between the oscillating tip of the device and tissue. The amount of mechanical energy applied to the tissue is adjusted by varying the length of the excursion of the blade, which ranges between 50 and 100 µm. A larger amount of blade excursion results in more rapid cutting and less thermal spread, but also minimizes hemostasis. A shorter blade excursion results in less cutting and a greater degree of collateral thermal injury, which results in better hemostasis. Not only the excursion of the blade but also the lifting and tensioning of the tissue causes a difference in mechanical energy; therefore, this device would make a difference depending on the surgeon. Prolonged or inappropriate activation can cause contact tissue damage or blade fractures.

When handling ultrasound energy devices, surgeons should always focus on making contact with vital structures. Ultrasound energy devices have a concerned phenomenon, so-called “*cavitation*”, that it may damage a distant position different from the target tissue. However, no abnormal findings are found unless it is close to 0.1 mm [20]. The key feature is that it cuts at the tip (Fig. 4), and the cutting speed is faster than that of advanced bipolar devices [19]. There is no need to apply a dispersive electrode, and the absence of an electrical current passing through the patient’s body eliminates the complications of monopolar devices.

## Sealing effect of advanced energy devices

Advanced bipolar devices and ultrasonic energy devices have advantages, disadvantages, and commonalities (Table 2). Therefore, it is important to use them safely and understand their principles and characteristics.

**Table 2:** Comparison of advanced bipolar devices and ultrasonic energy devices

	Advanced bipolar	Ultrasonic energy
Sealing effect	Good	Good
Cutting effect	From the bottom to a short distance before the tip	From the bottom to the tip
Activation speed	Slower	Faster
Device temperature	Lower	Higher
Lateral thermal spread	Wider	Narrower
Tip form	Both side same	Attention to the active blade
Handling	No activation unless sandwiching	Activation without sandwiching
Cost	Expensive	Expensive

Various reports on the sealing effect of advanced energy devices exist. The mean burst pressure of the small pulmonary arteries is 4.3-fold lower after sealing than after ligation and 6.4-fold less after ligation of thick pulmonary arteries with LigaSure™. Sealed pulmonary arteries > 5 mm in diameter had a burst pressure that was 50% less than that of smaller arteries. Histologic examination after sealing demonstrated only fusion of the adventitia, and the intima and media were replaced and invaginated into the vessel lumen [21]. The EnSeal™ device resulted in a pressure tolerance of > 75 mmHg in the acute phase [22]. The mean bursting pressure with Harmonic™ was equal to or greater than that of a vascular stapler in a simulated ex vivo model [23]. Advanced energy devices provide a seal that was superphysiologic in that all burst pressures were > 250 mm Hg [24]. The sealing effect of the ultrasound energy device is similar to that of advanced bipolar devices [23–26]. The sealing time of the ultrasound energy device was significantly shorter than that of advanced bipolar devices [26].

A prospective study demonstrated that LigaSure™ sealing without reinforcement allowed secure treatment during lung resection of pulmonary arteries as large as 5 mm in diameter and pulmonary veins as large as 7 mm [27]. This study reported a postoperative hemorrhage that occurred in the case of a pulmonary artery as large as 7 mm without any additional reinforcement. Thus, pulmonary arteries > 5 mm are appropriate for central treatment such as clipping and ligation on the hilar side [28].

Overall, sealing using advanced energy devices is safer to use in vessels of 5 mm or less. As it is difficult to accurately measure the vessel diameter intraoperatively, it is challenging to use vessel diameter empirically based on visual information with reference to the diameter of the device. Any force in the sealing area may cause bleeding. In addition, arteries, veins, and lymphatic vessels have histological differences. Moreover, patients with diabetes, vasculitis, and arteriosclerosis have different tissues. If bleeding is a concern, ligation should be performed, or an automatic suture device should be used instead of an advanced bipolar device alone.

## Energy devices in lung cancer surgery

Pulmonary resection requires a skin incision, separation of the subcutaneous tissue, and access to the thoracic cavity. In addition, anatomical lung resection requires cutting of pulmonary veins, pulmonary arteries, bronchi, and interlobar or intersegment divisions. In the case of lung cancer, dissection of the hilar and mediastinal lymph nodes is also performed. Since energy devices are used in each of these processes, their selection criteria, merits, and precautions are described as follows (Table 3).

**Table 3:** Procedure options in lung cancer surgery

Organs	Options	Inappropriate/caution
Skin	Cold scalpel	
Vessels		
Pulmonary vessels		
≥ 8 mm	L, St	Energy device
6–7 mm	L, St, advanced energy device with clip or ligation	Energy device without clip or ligation
≤ 5 mm	L, St, advanced energy device with/without clip or ligation	
Bronchial artery	L, advanced energy device	
Lymphatic vessels		
Trachea/Bronchi	St, cold scalpel with suturing or ligation	Cutting with energy device, monopolar device on the stapler
Lung parenchyma		
Partial lung resection Interlobar fissure division	St, suturing, advanced energy device for thin tissue	Monopolar device on the stapler
Intersegment division	St, suturing, energy devices	
Lymph node dissection		Monopolar and advanced bipolar devices use close to nerves

*L* ligation, *St* stapler

### Skin incision

Skin incisions are traditionally performed with a cold scalpel. Incisions performed with energy devices generally increase the risk of delayed wound healing and scarring, although this can be avoided with some devices and modes [29, 30].

### Subcutaneous tissue exfoliation

In VATS, the port is used, sparing the muscles; thus, muscle damages are minimal. Contrarily, conventional thoracotomy requires amputation of the muscle. In a rabbit experiment, the outcomes of muscle tissue healing were poor in the coagulation mode compared with that in the cut mode and using cold scalpel [31]. Although the clinical outcomes are uncertain, it may be advisable to avoid cutting in the coagulation mode during thoracotomy.

### Pulmonary arteries and veins

Traditionally, pulmonary arteries and veins have been treated by ligation, but now it is possible to safely dissect them using staplers and advanced energy devices such as advanced bipolar devices and ultrasonic energy devices [32]. Neither the stapler nor advanced energy device depends on the operator skill, unlike ligation. Energy devices have the advantage of prevention of residual artificial material. Cutting small blood vessels with a stapler may cause oozing, whereas cutting large blood vessels with an advanced energy device increases the risk of severe bleeding. However, the diameter of the blood vessel has a substantial influence on the safe separation by the advanced energy device. There have been some reported experiments and clinical studies, but the backgrounds such as devices used, target blood vessels, and periods were different [23–26, 33]. Presently, advanced energy devices alone can be safely used for vessels sized ≤ 5 mm, regardless of whether they are pulmonary arteries or veins [23, 27]. If sized ≥ 6 mm, the pressure resistance will decrease; hence, it will be safer to secure the center with ligation or clip [21, 22, 28, 34]. Since precise measurement of the vessel diameter is not possible during surgery, it is desirable to refer to the shaft diameter or to use ligation or clip together instead of using the energy device alone in cases of anxiety or discomfort. Care must be exercised because the sealing site may collapse due to the contact with the cutoff end.

### Bronchi

The bronchi can be safely and easily dissected with a stapler; however, there are no reports on safe bronchial separation using energy devices [35]. Dissecting the bronchi with an energy device involves the risk of not only stump fistula but also airway burns and operating room fire [36]. Bronchial complications such as bronchopleural fistula and bleeding from the bronchial artery often need reoperation and are fatal [37–39]. Since the bronchial artery is the most common cause of bleeding after lung resection, it is necessary to fully confirm this artery during the operation [37, 40]. However, hemostasis of the bronchial stump bleeding with a monopolar cut is associated with a risk of bronchopleural fistula. Monopolar devices diverge current to the staple metal in all modes [5] (Ref; 4. The Art and Science of Monopolar Electrosurgery). Monopolar devices at any modes should not be used to stop bleeding around the bronchi, as they can cause delayed bronchial stump fistulas [18]. In case of bronchial stump bleeding, suturing and advanced energy devices are safer hemostasis options than monopolar devices.

### Lung parenchyma

A stapler is used in pulmonary resection, which can be safely used not only for pulmonary arteries, veins, and bronchi but also for the lung parenchyma. Less air leakage with powered-type stapler than with a manual type was recently reported [41–43].

However, a stapler is not always the best option in all cases. Staples are often used in partial lung resection and interlobar and intersegment divisions, but some facilities prefer energy devices. Device options include monopolar, bipolar, advanced bipolar, and ultrasonic energy devices, as well as laser. However, the optimal device, as compared with staplers, remains controversial.

### Partial lung resection

There are some reports on energy devices for partial lung resection reporting results and tissue damage. Partial lung resection is safer using LigaSure™ and Harmonic™ than using staples, and air tightness is reported to be equivalent [44–49]. LigaSure™ has been reported to cause less tissue damage than Harmonic™ or monopolar, but its clinical impact is uncertain [48, 49]. Postoperative air leak is thought to be influenced by the depth of the lung parenchyma that energy devices can access and its protein content. Laser partial lung resection is airtight at a depth of 1.5 cm but requires additional sutures at depths of ≥ 1.5 cm [49]. Although partial lung resection using an energy device is not obvious in cases with lung diseases such as emphysema and interstitial pneumonia, it is not realistic and uncertain. Partial lung resection mainly involves using a stapler, and an energy device is used when a deep margin cannot be secured. Additional sutures should be considered when using energy devices. However, one of the problems with using a stapler is high cost.

### Interlobar fissure division

A stapler is often used for interlobar fissure division, with an energy device being another option. The decision to use an energy device or stapler depends on tissue thickness; it is not necessary to use a stapler in cases of good interlobar fissure. It is controversial in cases with incomplete interlobar fissure. A stapler is commonly used, but energy devices are reportedly not inferior to staplers [50–52]. In addition to energy devices, a sealant may reduce postoperative air leaks and promote safer division [53].

### Intersegment division

As with interlobar fissure division, staplers are often used for intersegment division. Energy devices reportedly do not interfere with lung dilation and are superior to staplers in terms of preserving lung function [54, 55]. Currently, inter-segmental division with a stapler has no difference in lung function compared to energy device formation [56, 57], and is superior to energy device formation in that there are fewer complications such as air leaks [58, 59].

### Lymph node dissection

Lymph node dissection is performed by excising all tissue in the compartment surrounded by some anatomic tissue, such as the trachea, bronchus, superior vena cava, aorta, pericardium, and esophagus. Since blood vessels, lymph vessels, and nerves run along the tissues surrounding the lymph nodes, the main complications associated with dissection include recurrent laryngeal nerve palsy, chylothorax, and arrhythmia.

Chylothorax occurs after lung cancer surgery at a frequency of 0.25–3%, with risk factors being extended resection, right side resection, and systematic lymph node dissection. The causal relationship of postoperative chylothorax with histology and stage is controversial [60]. Chylothorax is a complication that should be avoided because it requires reoperation and prolonged hospitalization. Prevention of postoperative chylothorax involves proper sealing of lymphatic vessels. Both advanced bipolar devices and ultrasonic energy devices have sufficient pressure resistance for thoracic duct treatment and are considered clinically acceptable [61–63]. Compared with monopolar devices, LigaSure™ is reportedly effective in short operations with a short drainage period, and it is considered that the reliable sealing effect reduces drainage from lymphatic vessels [64].

The incidence of recurrent laryngeal nerve palsy after systematic lymph node dissection is 9.7% [65]. Among such cases of recurrent laryngeal nerve palsy, hoarseness improves in 72.7% over an average of 10 months. Recurrent laryngeal nerve palsy should be avoided as it can be fatal, affect the quality of life, cause aspiration pneumonia, and may last a lifetime as a sequela. Recovery is faster without the use of energy devices, even with recurrent laryngeal nerve palsy [66]. Contrarily, there is a report that there is no difference with or without the use of energy devices. The complication rate of recurrent laryngeal nerve palsy varies, depending on the anatomical position of the recurrent laryngeal nerve, the device used, its distance from the nerve, and each factor of activation time [19]. A comparison between the advanced bipolar device and the ultrasonic energy device revealed that the advanced bipolar device was hotter than the ultrasonic energy device; however, the lateral heat diffusion was higher with the advanced bipolar device, and the temperature increased even at a distance of 3 mm from the nerve. Nevertheless, as there is no difference in lateral heat diffusion between the two when activated within 3 s, it is considered that the difference between energy devices is minimized when activated for a short time. Therefore, it is important to use the recurrent laryngeal nerve at an appropriate distance from the nerve depending on the device and to minimize activation.

### Adhesions

Adhesion is a condition in which membranes that are usually separate adhere to each other due to inflammation. It obscures visible boundaries, making it difficult to understand the anatomical structure and thereby leading to unintentional damage to organs and complications.

In lung cancer surgery, severe adhesions were subdivided into three groups depending on the location: whole thoracic cavity, locally invasive, and vascular sheath adhesions. Chest ultrasound sonography is useful for assessing adhesions throughout the thoracic cavity [67, 68]. Cine MRI would be effective in imaging areas not accessible by ultrasonography. The presence or absence of adhesions is an important factor when deciding the approach for arthroscopic surgery.

Cases of vascular sheath adhesion tend to have similar intrathoracic lymph nodes, suggesting pneumoconiosis. The shape and appearance were amebiform, and the boundaries between the lymph node and pulmonary artery sheath were unclear. The pulmonary artery sheath was located between the adventitia and media because the lymph nodes soaked into the pulmonary artery sheath. Chemotherapy may have a risk of vascular sheath adhesions. Since thoracoscopic avulsion of the pulmonary artery was difficult and risky, thoracotomy would be safer.

Severe adhesions increase the risk of pulmonary fistula, bleeding, prolonged surgery time, and conversion to thoracotomy. There are no reports regarding the differences between energy devices for adhesion detachment and will depend on the surgeon’s choice and skill. Da Vinci is reportedly effective for peeling [69], possibly because CO_2_ insufflation improves the visibility of the peeling line.

### Usefulness of soft coagulation

**Fig. 5:**
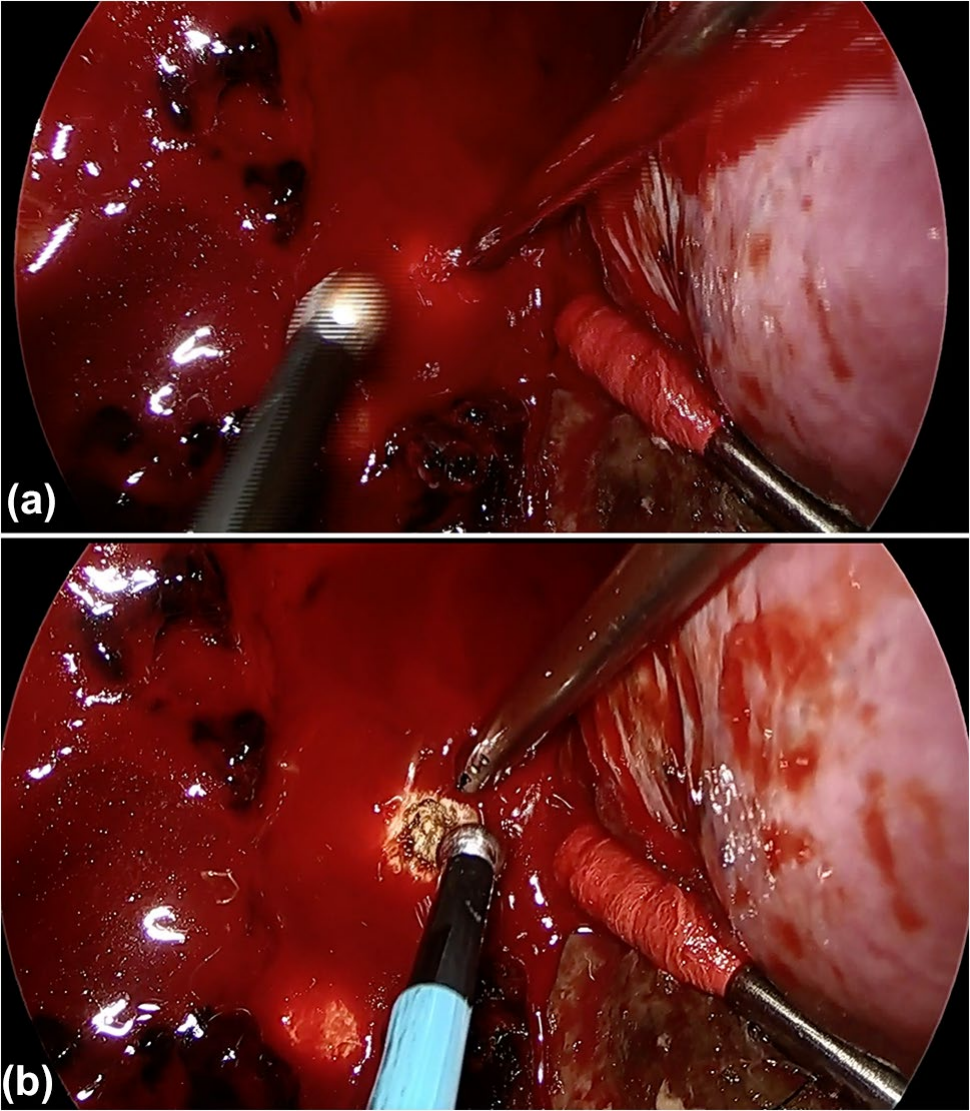
**a** A case of idiopathic hemothorax. Erupted bleeding was observed from the chest wall. **b** Soft coagulation with a ball-type electrode resulted in hemostasis

Soft coagulation is a relatively new mode designed for better hemostasis, and its principle is an unmodulated, very low voltage [14]. Thus, uniform protein coagulation is easily achieved, and the tissue closure (sealing) effect and hemostasis are better than that in other modes. In case of difficulty pinpointing or grasping the bleeding site, a ball electrode is useful. Bleeding from the chest wall, such as the intercostal arteries and veins, is pertinent (Fig. 5). Soft coagulation has also been reported to be useful for bleeding from the pulmonary artery [70]; however, it is not effective for critical bleeding. In addition to hemostasis, soft coagulation has been reported to affect pulmonary bulla coagulation, lung intersegment marking, and pulmonary resection [51, 71, 72]. Soft coagulation for pulmonary fistula closure is not effective in emphysema with low protein content and may lead to intractable pulmonary fistula when carbonized. As mentioned above, use around staples is not recommended because of the risk of intractable fistulas [5] (Ref; 4. The Art and Science of Monopolar Electrosurgery). It is safer to use soft coagulation as a supportive treatment in consideration of tissue conditions.

## Operating room fire risks

Operating room fires are a rare adverse event, estimated at 550–650 fires annually in the United States [73]. Ninety-five percent of cases are mild, but 5% are serious, with 20–30 cases annually leading to death. Seventy percent of ignition sources are electrosurgical equipment. Fires occur around the head, neck, face, or upper chest and in the airway [74, 75].

In lung cancer surgery, tracheoplasty, bronchoplasty, tracheostomy, and bronchoscopic procedures are associated with a risk of operating room fire. In tracheostomy, tracheoplasty, and bronchoplasty, a cold knife rather than an energy device should be used for incisions of the trachea and/or bronchus [36]. Oxygen in the airways can ignite. After the incision, an electrosurgical device should not be used because of the oxygen that is released into the surgical field. When oxygen is required, isolated lung ventilation or ventilating the area should be considered. To prevent operating room fires during oxygen administration, the lowest required concentration and flow rate of oxygen should be used, or intermittent rather than continuous administration should be considered.

Airway burns may also occur when using energy devices for cauterization and hemostasis of intra-airway tumors in bronchoscopic procedures. The main energy devices used for bronchoscopic procedures are lasers, argon plasma coagulation, polypectomy snare, and hot biopsy forceps. All these can cause ignition during treatment at high oxygen levels. If the oxygen concentration is 30% or more, there is a risk of ignition [76, 77], which is an important consideration in respiratory management. For airway bleeding, energy devices should not be used, and adrenaline-diluted saline and thrombin should be prioritized.

## Energy devices and basic principles of surgery

**Fig. 6:**
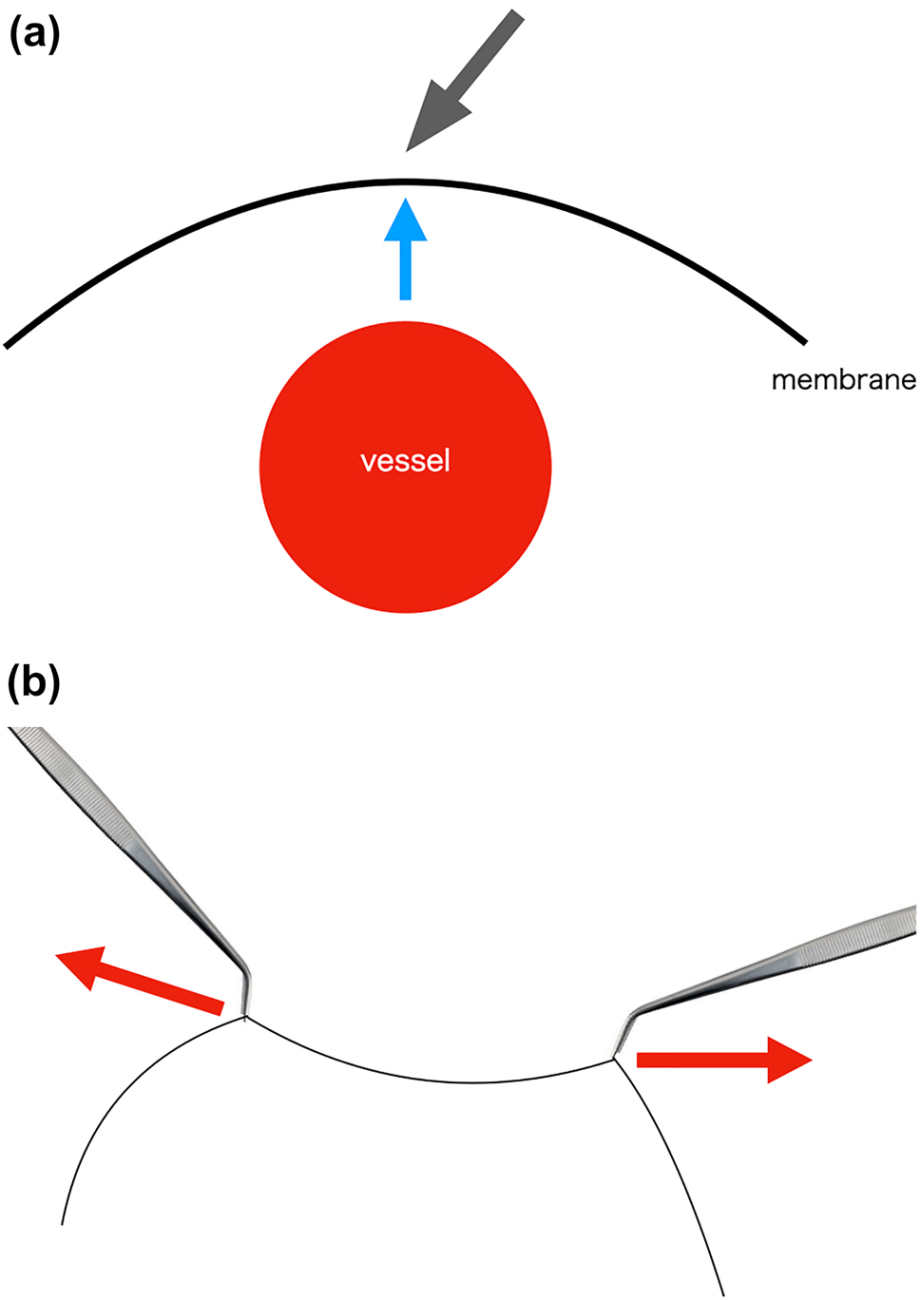
**a** “Off the ground” involves raising of the superficial tissue and keeping it away from the deep tissue to prevent damage to the deep tissue structures such as vessels or nerves. **b** “Counter traction involves” application of force in the opposite direction by the surgeon and assistant to apply tension to the grasped tissue

There is a time-tested adage as follows: “You cannot cut, what you cannot see.” A good surgery requires a simple maneuver that simultaneously promotes both adequate surgical site exposure and sufficient traction. This can be achieved by two principles: “off the ground” and “counter traction.” “Off the ground” involves a procedure that separates the targeted tissue from the deep tissue and protects the deep tissue. “Counter traction” involves a procedure wherein appropriate tension to the targeted tissue is applied in the opposite direction by the surgeon and assistant, thus facilitating appropriate incision of the target tissue. For the safe use of energy devices, both “off the ground” and “counter traction” need to be considered (Fig. 6). In particular, both principles should be consciously applied when using a spatula-type monopolar device. Depending on the surgical approach, different energy devices are useful. In thoracotomy and multiple-port VATS, “counter traction” and “off the ground” are possible at will, and a spatula-type monopolar device is appropriate. However, in uniportal VATS, an assistant cannot freely assist; therefore, the use of a spatula-type monopolar device is restricted [78]. Instead, the hook type is more convenient in uniportal VATS, because it allows the operator to implement the “off the ground” principle himself or herself. However, even with different surgical approaches, advanced bipolar devices and ultrasonic energy devices work on the sandwiched tissue, reducing awareness of “counter traction.”

## Conclusions

The present review examined energy devices, including monopolar devices, bipolar devices, and ultrasonic devices; electrosurgery-related injuries; fundamentals of electrosurgery; operating room fire risks; and basic principles of surgery. Understanding these energy devices can lead to more effective and safer use and can facilitate better decision-making regarding these energy devices in lung cancer surgery.

## Supplementary Information

Below is the link to the electronic supplementary material.

Supplementary file1 (DOCX 27 KB)

## Data Availability

The data that support the findings of this manuscript are available from the corresponding author, Takahiro Homma, upon reasonable request.

## References

[1] Ginsberg RJ, Rubinstein LV (1995). Randomized trial of lobectomy versus limited resection for T1 N0 non-small cell lung cancer. Lung Cancer Study Group. Ann Thorac Surg.

[2] Sihoe ADL (2019). Uniportal lung cancer surgery: state of the evidence. Ann Thorac Surg.

[3] Veronesi G, Novellis P, Voulaz E, Alloisio M (2016). Robot-assisted surgery for lung cancer: state of the art and perspectives. Lung Cancer.

[4] Licht PB (2016). Subxiphoid uniportal lobectomy. Eur J Cardiothorac Surg.

[5] Feldman LS, Fuchshuber PR, Jones DB (2021). The SAGES manual on the fundamental use of surgical energy (FUSE).

[6] Watanabe Y, Kurashima Y, Madani A, Feldman LS, Ishida M, Oshita A (2016). Surgeons have knowledge gaps in the safe use of energy devices: a multicenter cross-sectional study. Surg Endosc.

[7] Lee J (2002). Update on electrosurgery Outpatient Surg.

[8] Nduka CC, Super PA, Monson JR, Darzi AW (1994). Cause and prevention of electrosurgical injuries in laparoscopy. J Am Coll Surg.

[9] Tucker RD (1995). Laparoscopic electrosurgical injuries: survey results and their implications. Surg Laparosc Endosc.

[10] Pharmaceuticals and Medical Devices Agency (PMDA). Safety information. https://www.pmda.go.jp/english/pnavi_e-02.html. Accssed 28 Feb 2021.

[11] Nishihara Y, Watanabe Y, Homma T, Kishiki T, Isobe Y (2021). Energy device education utilizing the fundamental use of surgical energy (FUSE) program. Operation.

[12] Homma T, Watanabe Y (2019). Basic knowledge and safe use of electrosurgery. J Clin Surg.

[13] Ohm GS. Die galvanische Kette, mathematics bearbeitet. Berlin, Germany: Bei T.H. Riemann, 1827. 10.5479/sil.354716.39088005838644. Accessed 28 Feb 2021.

[14] Watanabe Y, Fuchshuber P, Homma T, Bilgic E, Madani A, Hiki N (2020). An unmodulated very-low-voltage electrosurgical technology creates predictable and ultimate tissue coagulation: from experimental data to clinical use. Surg Innov.

[15] Bae HS, Lee MY, Park JU (2018). Intraoperative burn from a grounding pad of electrosurgical device during breast surgery: a CARE-compliant case report. Medicine (Baltimore).

[16] Montero PN, Robinson TN, Weaver JS, Stiegmann GV (2010). Insulation failure in laparoscopic instruments. Surg Endosc.

[17] Espada M, Munoz R, Noble BN, Magrina JF (2011). Insulation failure in robotic and laparoscopic instrumentation: a prospective evaluation. Am J Obstet Gynecol.

[18] Shibano T, Endo S, Otani S, Nakano T (2015). Incidental bronchial injury by soft coagulation. J Thorac Dis.

[19] Hayami M, Watanabe M, Mine S, Imamura Y, Okamura A, Yuda M (2019). Lateral thermal spread induced by energy devices: a porcine model to evaluate the influence on the recurrent laryngeal nerve. Surg Endosc.

[20] Hashimoto S, Tatsuoka H, Matsubara H, Yamaguchi T, Hayashi H (2010). Analysis of tissue damage by ultrasonically activated device. J Jpn Soc Endosc Surg.

[21] Lesser TG, Wolfram F, Boltze C (2013). Sealing of pulmonary arteries with LigaSure: in vivo and ex vivo examinations. J Thorac Cardiovasc Surg.

[22] Yamada T, Sowa T, Bando T, Date H (2016). Experimental study in pulmonary artery sealing with a vessel-sealing device. Asian Cardiovasc Thorac Ann.

[23] Liberman M, Khereba M, Nasir B, Goudie E, Danino A, Giot JP (2015). Pulmonary artery sealing using the HARMONIC ACE+ shears for video-assisted thoracoscopic surgery lobectomy. Ann Thorac Surg.

[24] Okhunov Z, Yoon R, Lusch A, Spradling K, Suarez M, Kaler KS (2018). Evaluation and comparison of contemporary energy-based surgical vessel sealing devices. J Endourol.

[25] Newcomb WL, Hope WW, Schmelzer TM, Heath JJ, Norton HJ, Lincourt AE (2009). Comparison of blood vessel sealing among new electrosurgical and ultrasonic devices. Surg Endosc..

[26] Person B, Vivas DA, Ruiz D, Talcott M, Coad JE, Wexner SD (2008). Comparison of four energy-based vascular sealing and cutting instruments: a porcine model. Surg Endosc.

[27] Okada M, Miyata Y, Takamochi K, Tsutani Y, Oh S, Suzuki K (2019). Prospective feasibility study of sealing pulmonary vessels with energy in lung surgery. J Thorac Cardiovasc Surg.

[28] Lucchi M, Duranti L, Melfi F, Mussi A (2010). Polymer self-locking clips for vascular control during minimally invasive pulmonary lobectomies. J Thorac Cardiovasc Surg.

[29] Lee BJ, Marks M, Smith DP, Hodges-Savola CA, Mischke JM, Lewis RD (2014). Advanced cutting effect system versus cold steel scalpel: comparative wound healing and scar formation in targeted surgical applications. Plast Reconstr Surg Glob Open.

[30] Wu AY, Baldwin TJ, Patel BC, Clymer JW, Lewis RD (2017). Healing comparison of porcine cutaneous incisions made with cold steel scalpel, standard electrosurgical blade, and a novel tissue dissector. Med Res Innov.

[31] Bowers CA, Burns G, Salzman KL, McGill LD, Macdonald JD (2014). Comparison of tissue effects in rabbit muscle of surgical dissection devices. Int J Surg.

[32] Yano M, Takao M, Fujinaga T, Arimura T, Fukai I, Ota S (2013). Adverse events of pulmonary vascular stapling in thoracic surgery. Interact Cardiovasc Thorac Surg.

[33] Liberman M, Khereba M, Goudie E, Kazakov J, Thiffault V, Lafontaine E (2014). Pilot study of pulmonary arterial branch sealing using energy devices in an ex vivo model. J Thorac Cardiovasc Surg.

[34] Harold KL, Pollinger H, Matthews BD, Kercher KW, Sing RF, Heniford BT (2003). Comparison of ultrasonic energy, bipolar thermal energy, and vascular clips for the hemostasis of small-, medium-, and large-sized arteries. Surg Endosc.

[35] Yano M, Yokoi K, Numanami H, Kondo R, Ohde Y, Sugaya M (2014). Complications of bronchial stapling in thoracic surgery. World J Surg.

[36] Gorphe P, Sarfati B, Janot F, Bourgain JL, Motamed C, Blot F (2014). Airway fire during tracheostomy. Eur Ann Otorhinolaryngol Head Neck Dis.

[37] Sirbu H, Busch T, Aleksic I, Lotfi S, Ruschewski W, Dalichau H (1999). Chest re-exploration for complications after lung surgery. Thorac Cardiovasc Surg.

[38] Sirbu H, Busch T, Aleksic I, Schreiner W, Oster O, Dalichau H (2001). Bronchopleural fistula in the surgery of non-small cell lung cancer: incidence, risk factors, and management. Ann Thorac Cardiovasc Surg.

[39] Yang Y, Gao W, Zhao H, Yang Y, Shi J, Sun Y (2016). Risk factors and consequences of perioperative reoperation in patients undergoing pulmonary resection surgery. Surgery.

[40] Yano M, Numanami H, Akiyama T, Taguchi R, Furuta C, Haniuda M (2019). Reoperation for postoperative bleeding following pulmonary resection: a report of a single-center experience. Gen Thorac Cardiovasc Surg.

[41] Qiu B, Yan W, Chen K, Fu X, Hu J, Gao S (2016). A multi-center evaluation of a powered surgical stapler in video-assisted thoracoscopic lung resection procedures in China. J Thorac Dis.

[42] Shigeeda W, Deguchi H, Tomoyasu M, Kudo S, Kaneko Y, Kanno H (2021). Utility of the powered stapler for radical pulmonary resection: a propensity score-matched analysis. Surg Today.

[43] Miller DL, Roy S, Kassis ES, Yadalam S, Ramisetti S, Johnston SS (2018). Impact of powered and tissue-specific endoscopic stapling technology on clinical and economic outcomes of video-assisted thoracic surgery lobectomy procedures: a retrospective, observational study. Adv Ther.

[44] Kovács O, Szántó Z, Krasznai G, Herr G (2009). Comparing bipolar electrothermal device and endostapler in endoscopic lung wedge resection. Interact Cardiovasc Thorac Surg.

[45] Samancilar O, Cakan A, Cetin Y, Cagirici U, Veral A, Zeytunlu M (2007). Comparison of the harmonic scalpel and the ultrasonic surgical aspirator in subsegmental lung resections: an experimental study. Thorac Cardiovasc Surg.

[46] Shigemura N, Akashi A, Nakagiri T, Ohta M, Matsuda H (2004). A new tissue-sealing technique using the Ligasure system for nonanatomical pulmonary resection: preliminary results of sutureless and stapleless thoracoscopic surgery. Ann Thorac Surg.

[47] Cakan A, Yoldas B, Samancilar O, Ertugrul V, Turhan K, Cagirici U (2009). Ligasure vessel sealing system versus harmonic scalpel for sutureless nonanatomical pulmonary resections in a rabbit model. Which one is safer. Eur Surg Res.

[48] Sugimoto S, Toyooka S, Iga N, Furukawa M, Sugimoto R, Shien K (2014). Use of a vessel sealing system versus conventional electrocautery for lung parenchymal resection: a comparison of the clinicopathological outcomes in porcine lungs. Surg Today.

[49] Kirschbaum A, Surowiec TM, Pehl A, Gockel A, Bartsch DK, Mirow N (2018). Suturing of the laser resection area is recommended over a depth of 2 cm in an experimental porcine lung model. J Thorac Dis.

[50] Sakuragi T, Takeda Y, Teishikata T, Sakoda K, Morita S (2013). Is bipolar thermofusion an acceptable option for unseparated interlobar fissure division in pulmonary lobectomy. Interact Cardiovasc Thorac Surg.

[51] Sakuragi T, Okazaki Y, Mitsuoka M, Yamasaki F, Masuda M, Mori D (2008). The utility of a reusable bipolar sealing instrument, BiClamp((R)), for pulmonary resection. Eur J Cardiothorac Surg.

[52] Marulli G, Droghetti A, Di Chiara F, Calabrese F, Rebusso A, Perissinotto E (2013). A prospective randomized trial comparing stapler and laser techniques for interlobar fissure completion during pulmonary lobectomy. Lasers Med Sci.

[53] Droghetti A, Schiavini A, Muriana P, Folloni A, Picarone M, Bonadiman C (2008). A prospective randomized trial comparing completion technique of fissures for lobectomy: stapler versus precision dissection and sealant. J Thorac Cardiovasc Surg.

[54] Yoshimoto K, Nomori H, Mori T, Ohba Y, Shiraishi K, Ikeda K (2011). Comparison of postoperative pulmonary function and air leakage between pleural closure vs. mesh-cover for intersegmental plane in segmentectomy. J Cardiothorac Surg.

[55] Asakura K, Izumi Y, Kohno M, Ohtsuka T, Okui M, Hashimoto K (2011). Effect of cutting technique at the intersegmental plane during segmentectomy on expansion of the preserved segment: comparison between staplers and scissors in ex vivo pig lung. Eur J Cardiothorac Surg.

[56] Tao H, Tanaka T, Hayashi T, Yoshida K, Furukawa M, Yoshiyama K (2016). Influence of stapling the intersegmental planes on lung volume and function after segmentectomy. Interact Cardiovasc Thorac Surg.

[57] Tao H, Hayashi M, Furukawa M, Miyazaki R, Yokoyama S, Hara A (2019). Influence of intersegmental plane size and segment division methods on preserved lung volume and function after pulmonary segmentectomy. Gen Thorac Cardiovasc Surg.

[58] Chen X, Jin R, Xiang J, Han D, Zhang Y, Li C (2020). Methods for dissecting intersegmental planes in segmentectomy: a randomized controlled trial. Ann Thorac Surg.

[59] Ohtsuka T, Goto T, Anraku M, Kohno M, Izumi Y, Horinouchi H (2012). Dissection of lung parenchyma using electrocautery is a safe and acceptable method for anatomical sublobar resection. J Cardiothorac Surg.

[60] Chen C, Wang Z, Hao J, Hao X, Zhou J, Chen N (2020). Chylothorax after lung cancer surgery: a key factor influencing prognosis and quality of life. Ann Thorac Cardiovasc Surg.

[61] Novitsky YW, Rosen MJ, Harrell AG, Sing RF, Kercher KW, Heniford BT (2005). Evaluation of the efficacy of the electrosurgical bipolar vessel sealer (LigaSure) devices in sealing lymphatic vessels. Surg Innov.

[62] Nakayama H, Ito H, Kato Y, Tsuboi M (2009). Ultrasonic scalpel for sealing of the thoracic duct: evaluation of effectiveness in an animal model. Interact Cardiovasc Thorac Surg.

[63] Kajiyama Y, Iwanuma Y, Tomita N, Amano T, Hattori K, Tsurumaru M (2005). Sealing the thoracic duct with ultrasonic coagulating shears. Hepatogastroenterology.

[64] Martucci N, Tracey M, La Rocca A, La Manna C, De Luca G, Rocco G (2015). A pilot prospective randomized, controlled trial comparing LigaSure™ tissue fusion technology with the ForceTriad™ energy platform to the electrosurgical pencil on rates of atrial fibrillation after pulmonary lobectomy and mediastinal lymphadenectomy. Eur J Cardiothorac Surg.

[65] Sano Y, Shigematsu H, Okazaki M, Sakao N, Mori Y, Yukumi S (2019). Hoarseness after radical surgery with systematic lymph node dissection for primary lung cancer. Eur J Cardiothorac Surg.

[66] Yu M, Ge M (2021). Non-energy devices to dissect recurrent laryngeal nerve lymph nodes of non-small cell lung cancer under video-assisted thoracic surgery. BMC Surg.

[67] Homma T, Ojima T, Yamamoto Y, Shimada Y, Akemoto Y, Kitamura N (2020). Utility of the sliding lung sign for the prediction of preoperative intrathoracic adhesions. J Thorac Dis.

[68] Homma T, Shimada Y, Tanabe K, Akemoto Y, Ojima T, Yamamoto Y (2021). Adverse factors and postoperative neuropathic pain in challenging video-assisted thoracoscopic surgery. Ann Palliat Med.

[69] Martens TP, Morgan JA, Hefti MM, Brunacci DA, Cheema FH, Kesava SK (2005). Adhesiolysis is facilitated by robotic technology in reoperative cardiac surgery. Ann Thorac Surg.

[70] Sakuragi T, Okazaki Y, Mitsuoka M, Itoh T (2008). Dramatic hemostasis of the transected pulmonary artery model using SOFT COAG electrosurgical output. Interact Cardiovasc Thorac Surg.

[71] Kataoka D, Tomita Y, Fukayama M, Kadokura M, Yamochi T, Ota H (2011). Clinical effect of the cauterization for emphysematous bulla. Kyobu Geka.

[72] Ueda Y, Nakagawa T, Toyazaki T, Chiba N, Gotoh M (2016). A technique for identification and resection of the intersegmental plane in thoracoscopic segmentectomy. J Jpn Assoc Chest Surg.

[73] Sankaranarayanan G, Wooley L, Hogg D, Dorozhkin D, Olasky J, Chauhan S (2018). Immersive virtual reality-based training improves response in a simulated operating room fire scenario. Surg Endosc.

[74] Joint Commission Online. FDA issues guidance on how to prevent surgical fires. June 6, 2018. https://www.jointcommission.org/assets/1/23/JC_Online_June_6.pdf. Accessed 28 Feb 2021.

[75] ECRI Institute (2009). New clinical guide to surgical fire prevention Patients can catch fire—here's how to keep them safer. Health Devices.

[76] Jones TS, Black IH, Robinson TN, Jones EL (2019). Operating room fires. Anesthesiology.

[77] ECRI. Surgical fire prevention. https://www.ecri.org/Accident_Investigation/Pages/Surgical-Fire-Prevention.aspx. Accessed 28 Feb 2021.

[78] Liu C, Ma L, Guo C, Liu L (2016). Non-grasping en bloc mediastinal lymph node dissection through uniportal video-assisted thoracic surgery for lung cancer surgery. J Thorac Dis.

