# Supplementary material for: Advances and safe use of energy devices in lung cancer surgery
Source: Gen Thorac Cardiovasc Surg. 2022 Feb 2;70(3):207–18. doi: 10.1007/s11748-022-01775-w (PMC8881425; doi:10.1007/s11748-022-01775-w)
Supplement: Supplementary file 1 — Supplementary file1 (DOCX 27 KB) [file 11748_2022_1775_MOESM1_ESM.docx]

Supplementary Methods

To identify the effects and adverse events of energy devices, the PubMed, Medline, Scopus and Web of Sciences databases were searched electronically from their inception up to 16 April 2021 utilizing Boolean operators and combinations of word variants for ‘energy device’, ‘electrosurgery’, ‘’electrocautery’, ‘radiofrequency ablation’, ‘monopolar’, ‘monopolar devices’, ‘monopolar instruments’, ‘bipolar’, ‘bipolar devices’, ‘bipolar instruments’, ‘advanced bipolar devices’, ‘LigaSure’, ‘EnSeal’, ‘BiClamp’, ‘ultrasonic’, ‘ultrasonic energy devices’, ‘ultrasonic dissector’, ‘ultrasonic shears’, ‘Harmonic’, ‘Sonicision’, ‘staple’, ‘stapler’, ‘auto-suture’, ‘endostapler’, ‘staple line’, ‘vessel sealer’, ‘vessel sealing‘, ‘sealing’, ‘hemostasis’, ‘thermal spread’, ‘thermal change‘, ‘thermal injury’, ‘mode’, ‘cautery’, ‘cut’, ‘fulguration’, ‘coagulation’, ‘coag’, ‘soft coagulation’, ‘low-voltage’, ‘electrosurgical generator‘, ‘electrosurgical unit’, ‘modern ESU’, ‘duty cycle’, ‘adverse events’, ‘safety’, ‘electrosurgical injury’, ‘electrosurgery-related injury’, ’electrode-related injury’, ‘energy device failure’, ‘insulation failure’, ‘insulation breaks’, ‘active electrode’, ‘dispersive electrodes’, ‘fires’, ‘laser’, ‘operating room fire’, ‘surgical fire’, ‘operating room’, ‘surgery’, ‘thoracotomy’, ‘video-assisted thoracic surgery’, ‘video-assisted thoracoscopic surgery’, ‘VATS’, ‘minimally invasive surgery’, ‘uniport’, ‘uniportal’, ‘robot’, ‘robotic’, ‘robot-assisted surgery’, ‘subxiphoid’, ‘FUSE’, ‘skin incision’, ‘cold scalpel’, ‘cold knife’, ‘muscle’, ‘healing, scar’, ‘pulmonary artery’, ‘pulmonary vein’, ‘blood vessel’, ‘bleeding’, ‘bronchi’, ‘bronchus’, ‘lung’, ‘lung parenchyma’, ‘lung resection’, ‘partial resection’, ‘partial lung resection’, ‘segmentectomy’, ‘sublobar resection’, ‘lobectomy’, ‘interlobar fissure’, ‘intersegment division’, ‘air leak’, ‘air leakage’, ‘bronchopleural fistula’, ‘fistula’, ‘lymph node’, ‘dissection’, ‘lymphadenectomy’, ‘chylothorax’, ‘arrhythmia’, ‘recurrent nerve’, ‘recurrent laryngeal nerve’, ‘recurrent nerve palsy’, ‘recurrent laryngeal nerve palsy’, ‘hoarseness’, ‘thoracic duct’, ‘lymphatic vessel’, ‘adhesion’, ‘intrathoracic adhesion’, ‘adhesiolysis’, ‘complication’, ‘postoperative complications’, and ‘lung cancer‘. Papers published until the date of the review that contained these terms in the abstract were selected.
